# Laparoscopic subtotal gastrectomy in case of large subcardial GISTs

**DOI:** 10.1093/jscr/rjac396

**Published:** 2022-08-30

**Authors:** Sorin Cimpean, Flamand Francois, Mihai Stefan Muresan

**Affiliations:** General Surgery, South Iris Hospitals, Brussels, Belgium; General Surgery, Clinique Notre Dame de Grace, Charleroi, Belgium; General Surgery, Medicover Hospital, Cluj-Napoca, Romania

**Keywords:** gastrectomy, GIST, laparoscopy

## Abstract

The gastro-intestinal stromal tumours (GISTs) are rare mesenchymal tumours that occur mostly in the stomach. The treatment is usually a limited resection, which is performed by an endoscopy or by a surgical approach. In case of metastasis of the disease proven found during the assessment, the treatment is usually limited to chemotherapy without a radical cure. We report a case of a large (9 cm) subcardial GIST that we treated by laparoscopic subtotal gastrectomy due to the size and the location of the tumour. The laparoscopic resection is shown to be superior in perioperative outcomes compared to open surgery even for large lesions. In unfavourable locations such as in cardia lesions, transgastric, partial or extended gastric resections must be evaluated to avoid functional sequelae and post-operative morbidity.

## INTRODUCTION

The gastro-intestinal stromal tumour (GIST) is a rare mesenchymal tumour that occurs on the digestive tract. GIST occurs mostly on the stomach (55–60%), small intestine (30%), colon and rectum (10%) and the oesophagus (3%) [1, 2].

The malignant potential mostly depends on tumour size and mitotic activity. The lymph node involvement is rare [3]. Gastric GISTs present a better survival than small intestinal GISTs. Gastric GISTs <5 cm are usually benign, and many tumours between 5 and 10 cm with low mitotic activity had a good prognosis [4]. If the tumour does not present a loco-regional lymph node invasion, the 5-year survival rate is 93%. Extensive lymphadenectomy, as in case of gastric adenocarcinoma, is not necessary for these mesenchymal neoplasms [5].

We report in this publication a case of a large subcardial GIST tumour with no lymph node involvement, which was treated by subtotal gastrectomy.

## CASE PRESENTATION

**Figure 1:**
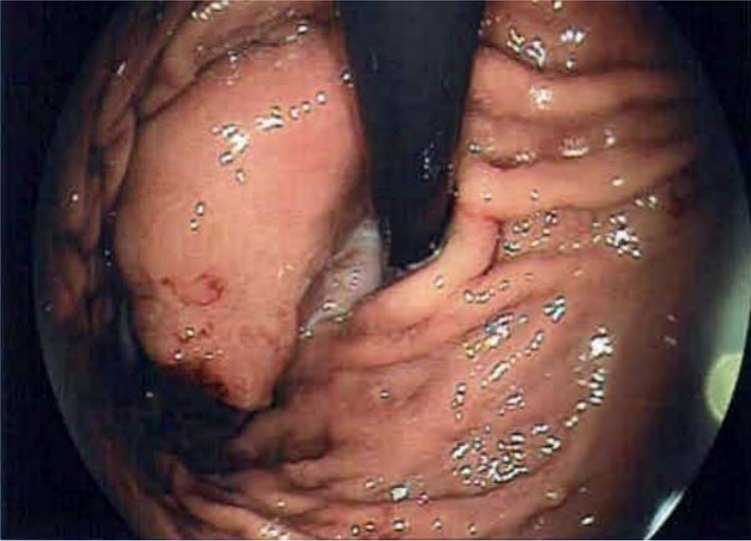
Endoscopy image that reveals the presence of the GIST in subcardial position.

A 66-year-old patient presented with episodic epigastric pain and a sensation of bloating in the gastro-enterology unit for an assessment. No associated symptoms like the loss of appetite or weight loss were described. The patient did not present with a family history of malignancy. The gastroscopy revealed a pericardial mass with extension on the gastric fundus (Fig. 1).

The biopsies revealed a GIST tumour with cells that express CD34, DOG1 and CD117 antibodies and for cytokeratin AE1 AE3 and three mitoses observed.

**Figure 2:**
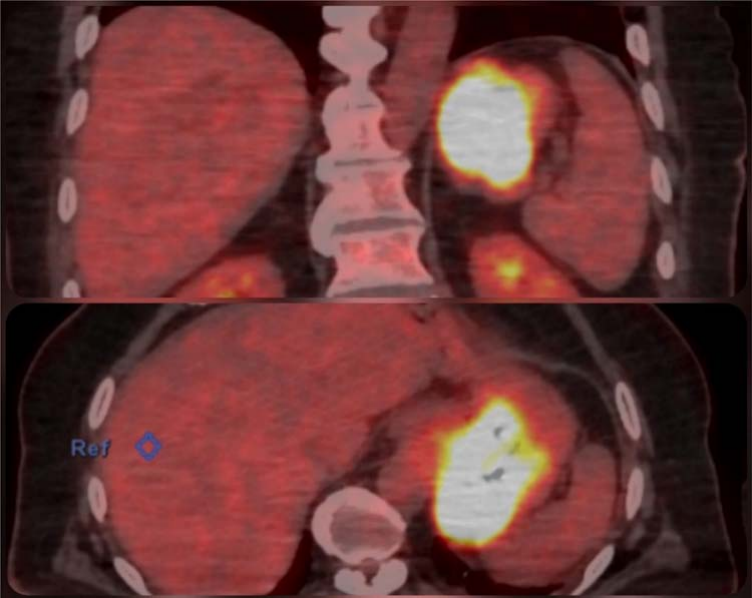
PET-CT image who shows the localisation of the GIST.

An abdominal computed tomography (CT) scan found a 9 × 8 cm subcardial lesion. The positron emission tomography (PET)-CT found a hypermetabolism on the gastric lesion with SUV max 21,5 and no locoregional hypermetabolic lymph nodes (Fig. 2).

The medical history of the patient revealed diabetes type 2 and a triple coronary bypass surgery that was performed in July 2015.

Due to the close proximity with the oeso-gastric junction, we decided to perform a subtotal gastrectomy with Roux-en-Y reconstruction.

## OPERATIVE TECHNIQUE

**Figure 3:**
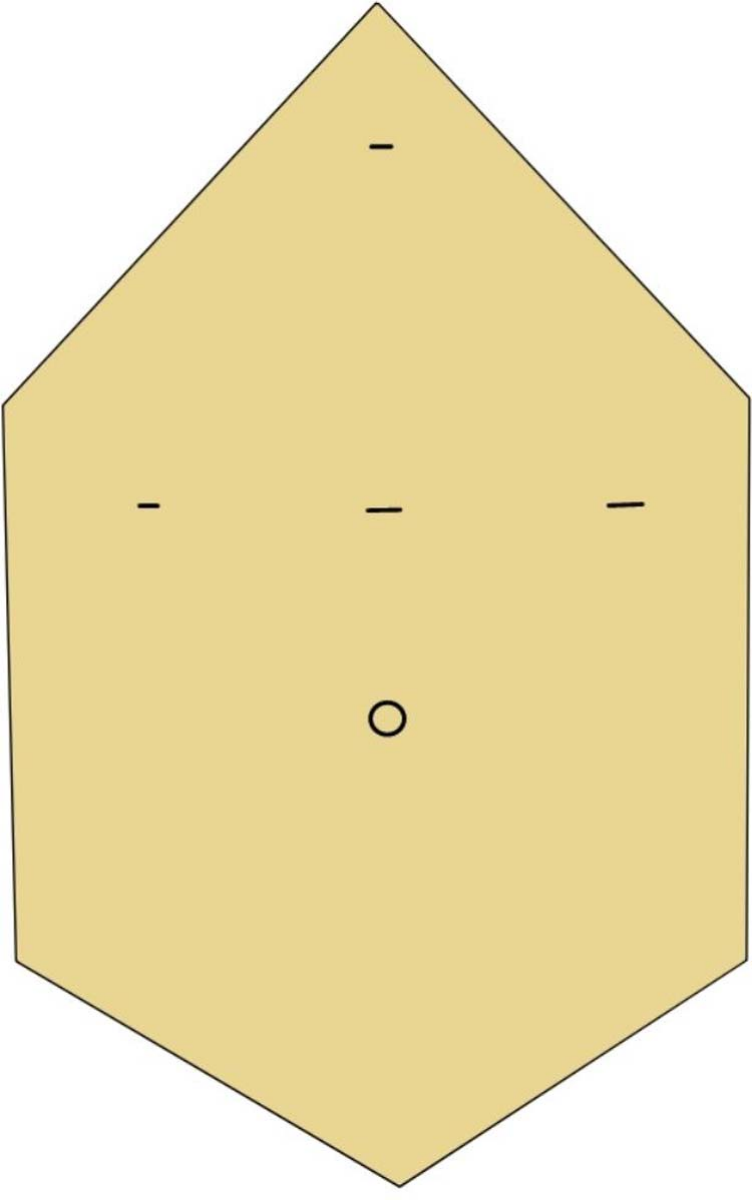
Trocars disposition.

Four trocars were placed (Fig. 3). The exploration found the large gastric GIST. The gastrocolic ligament was sectioned, and the retro-gastric access was performed. The right omental vessels and the left and right gastric vessels were clipped and sectioned. The capsular splenic injury during tumour mobilization required splenectomy for haemostasis.

**Figure 4:**
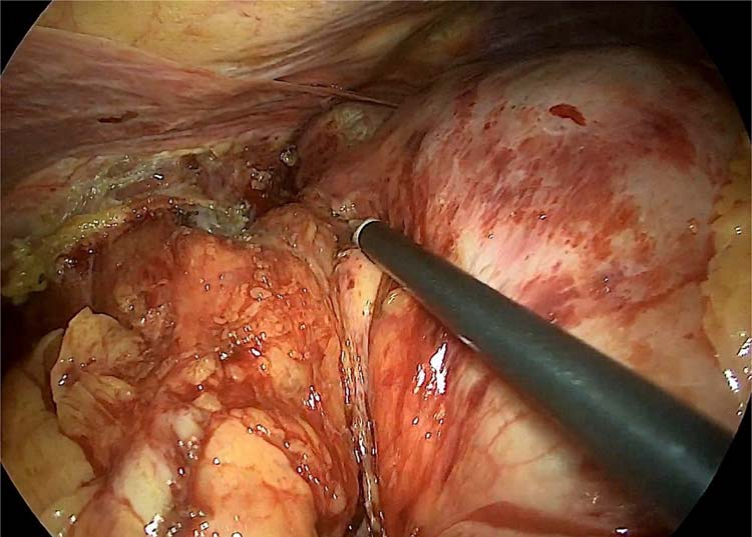
Intraoperative image that reveals the hiatal dissection and the proximity of the tumour with the cardia.

The hiatal region was dissected (Fig. 4). The gastric section was performed at the level of the pyloric antrum and the stomach at the level of the oeso-gastric junction. An intraoperative anatomopathological examination on the oesophageal section did not find any malignancy. A latero-lateral semi-mechanical gastrojejunal anastomosis was performed.

**Figure 5:**
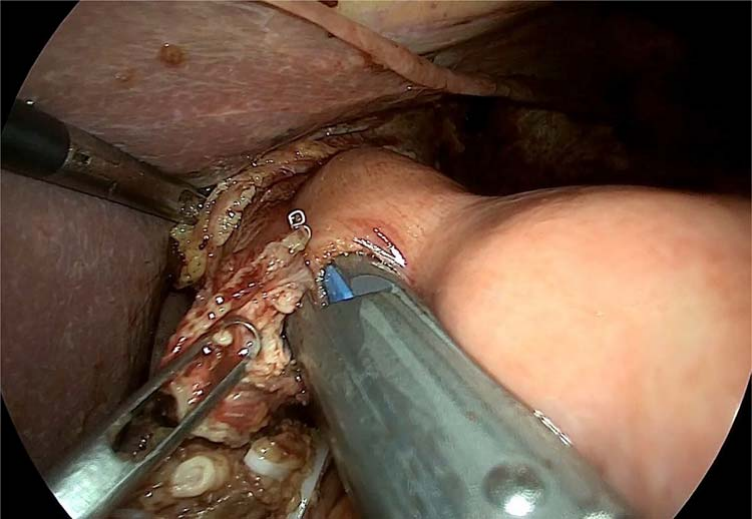
Intraoperative image that reveals the confection of oeso-jejunal anastomosis.

**Figure 6:**
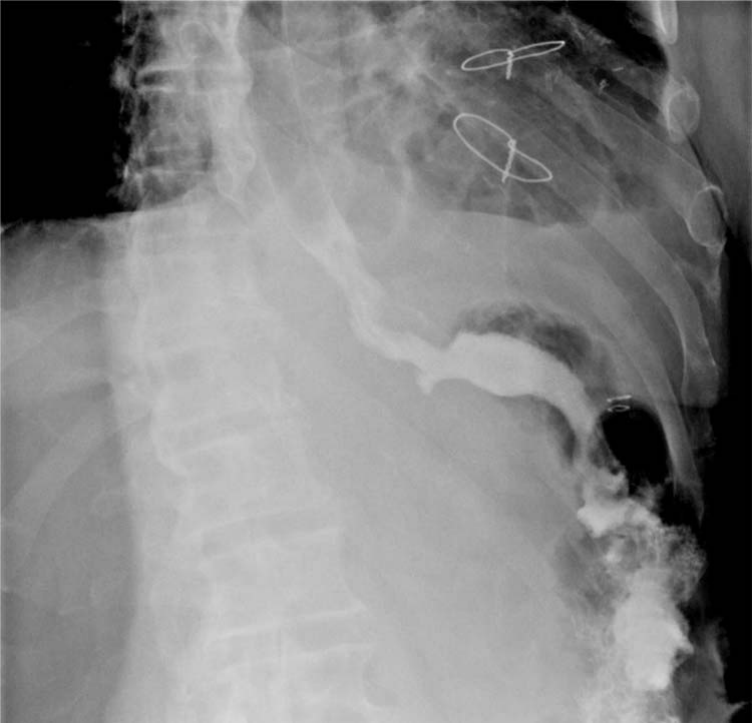
Oeso-jejunal post-operative transit.

A linear semi-mechanical latero-jejunal jejuno-jejunal anastomosis was performed at 60 cm from the oeso-gastric anastomosis (Fig. 5). The mesenteric space and Petersen’s space were closed using non-resorbable stitches. The spleen and the stomach were removed by a Pfannenstiel incision. A drain was left in place near the oeso-jejunal anastomosis. An oeso-jejunal transit was performed at the fifth post-operative day, which showed no signs of leakage (Fig. 6). The progressive oral alimentation was resumed.

The post-operative course was uneventful. The patient was discharged on the tenth post-operative day.

The histopathological exam found a ‘spindle cells’-type GIST with high mitotic rate (eight mitoses/5 mm [2]) and no lymph node involvement. The gastric margins were free of tumour. The immunohistochemical profile revealed the presence of CD117: ++ and DOG1: ++.

## DISCUSSIONS

The median age of diagnosis for GIST is 60 years, with the annual incidence estimated at 10–20 cases per million [6]. These tumours are asymptomatic in 22–31% of cases. The most common symptom is gastrointestinal bleeding that occurs in 52–54.5% of cases [7]. In our case, the patient presented with non-specific abdominal symptoms, with incidental discovery, despite the size of the mass and the proximity with the cardia.

The morphological includes spindle cell type (70%), epithelioid cell type (20%) or mixed type (10%). Ninety-five percent of GISTs are positive for KIT (CD117) and/or discovered on GIST-1 (DOG1), and 70% are positive for CD34 [8]. GIST with KIT exon 11 mutation presents a higher predisposition to liver metastasis [9]. Also, a high mitotic rate of ≥6 mitoses per 5 mm [2] presents an aggressive behaviour. The most common metastatic sites of GISTs are of the liver (65%) and peritoneum (21%); GISTs rarely metastasize to lymph nodes (6%), bone (6%) or lung (2%) [10].

The unfavourable locations for the surgical treatment are as follows: gastro-oesophageal junction, lesser curvature of gastric body, posterior wall of gastric body and gastric antrum [11]. For GIST at the gastro-oesophageal junction, enucleation and adjuvant therapies are described as alternatives to avoid the morbidity and mortality associated with oesophageal and oesophagogastric resections [12].

Imatinib, a competitive inhibitor of KIT tyrosine kinase, is effective in advanced GISTs [9]. The presence of metastasis are usually treated by chemotherapy without radical cure.

The prognosis of a GIST is ameliorated with early diagnosis and R0 resection [13]. R1 resections may be a solution when R0 resections involve significant functional sequelae and important morbidity. In the case of R1 resection, the overall survival rate is not well defined [14].

In literature, the laparoscopic resection is shown to be superior in perioperative outcomes compared to open surgery even for large lesions. In the case of partial gastrectomy, the combination of laparoscopy and endoscopy allows the complete resection of the GIST and also avoids any gastric deformation or stenosis with functional sequelae [15]. The transgastric transabdominal resection is a solution for subcardial lesions <4 cm.

Usually, the presence of gastric GIST allows a limited gastric resection. With the multiple techniques available, the treatment must be adapted for each case. In our case, the patient presented a large tumoural lesion, in subcardial position, and with extension to the fundic area. To avoid a stenosis and functional problems, we decided to perform a subtotal gastrectomy by laparoscopy. Splenectomy was performed because of the tight adhesions of the GIST to the splenic capsule and the potential risk of injury to the tumour capsule. Splenectomy is not systematically associated and is not part of the treatment of gastric GIST.

The laparoscopic approach is feasible and allows a diminution of pain and a quick recovery. This approach allows to keep the tumours’ capsule intact even in the case of large lesions.

In our case, the patient presents a high risk of stratification (tumour size: 90 × 80 × 60 mm, high mitotic activity: eight mitoses/5 mm^2^ and no capsule rupture). Due to the high risk of progressive disease (%) assessed at 55%, the patient was placed on Imatinib chemotherapy for 3 years with abdominal CT or magnetic resonance imaging every 3–6 months during adjuvant therapy.

## CONCLUSION

Gastric GIST are rare tumoural lesions with low potential of lymph node dissemination. In unfavourable locations as in cardia lesions, the transgastric resection, partial gastrectomy or the subtotal gastrectomy must be evaluated to avoid functional sequelae.
